# Molecular Mechanisms Controlling Lymphatic Endothelial Junction Integrity

**DOI:** 10.3389/fcell.2020.627647

**Published:** 2021-01-14

**Authors:** Pieter R. Norden, Tsutomu Kume

**Affiliations:** Department of Medicine, Feinberg School of Medicine, Feinberg Cardiovascular and Renal Research Institute, Northwestern University, Chicago, IL, United States

**Keywords:** lymphatic vessel junction, button-like junction, zipper-like junction, VEGF signaling, RhoA/ROCK, Notch, FOXC, Angiopoietin

## Abstract

The lymphatic system is essential for lipid absorption/transport from the digestive system, maintenance of tissue fluid and protein homeostasis, and immune surveillance. Despite recent progress toward understanding the cellular and molecular mechanisms underlying the formation of the lymphatic vascular system, the nature of lymphatic vessel abnormalities and disease in humans is complex and poorly understood. The mature lymphatic vasculature forms a hierarchical network in which lymphatic endothelial cells (LECs) are joined by functionally specialized cell-cell junctions to maintain the integrity of lymphatic vessels. Blind-ended and highly permeable lymphatic capillaries drain interstitial fluid via discontinuous, button-like LEC junctions, whereas collecting lymphatic vessels, surrounded by intact basement membranes and lymphatic smooth muscle cells, have continuous, zipper-like LEC junctions to transport lymph to the blood circulatory system without leakage. In this review, we discuss the recent advances in our understanding of the mechanisms by which lymphatic button- and zipper-like junctions play critical roles in lymphatic permeability and function in a tissue- and organ-specific manner, including lacteals of the small intestine. We also provide current knowledge related to key pathways and factors such as VEGF and RhoA/ROCK signaling that control lymphatic endothelial cell junctional integrity.

## Formation of the Lymphatic Vascular System

**Figure 1:**
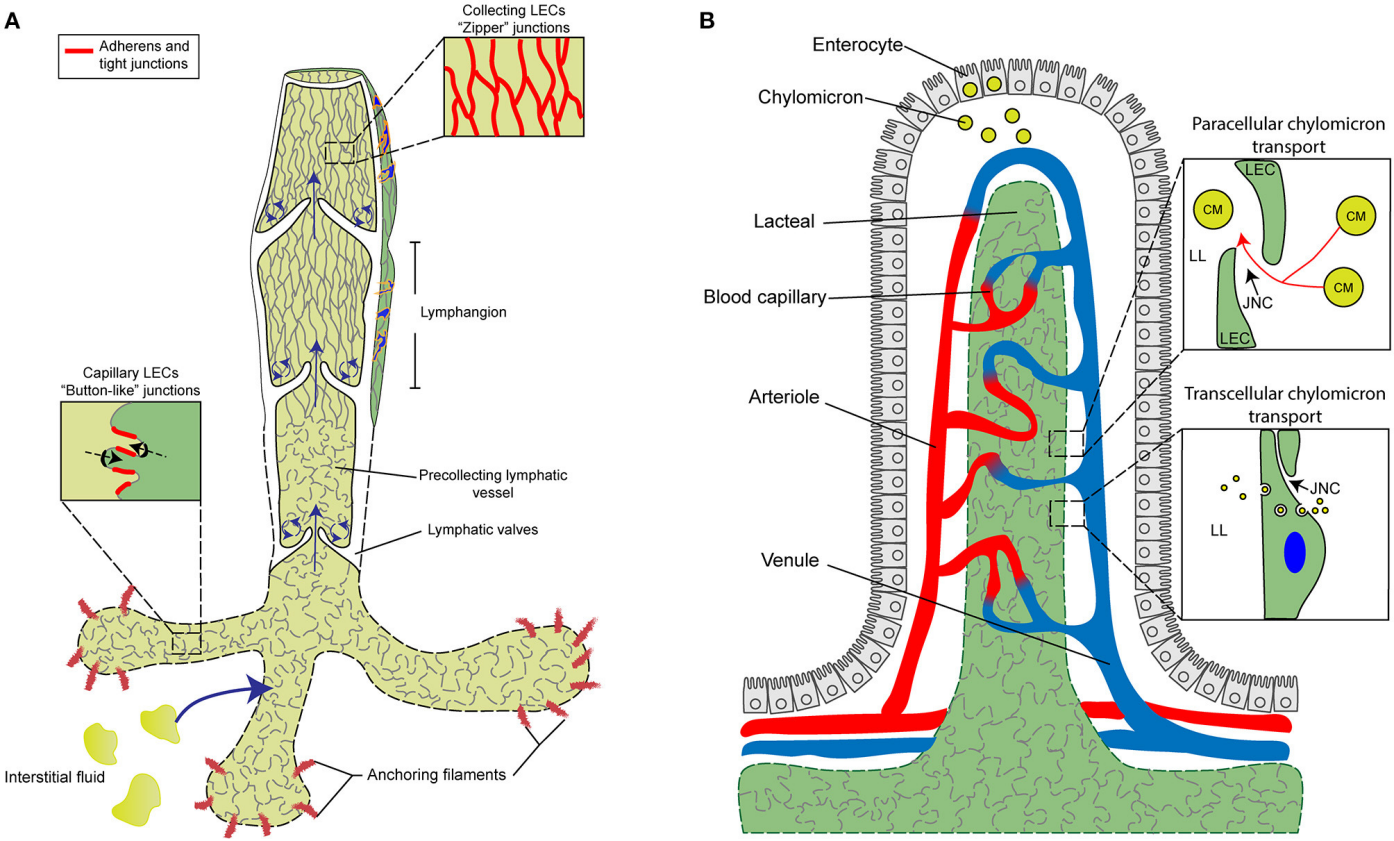
Characterization and function of cell junctions in the general lymphatic vasculature and lymphatic lacteals. **(A)** The general lymphatic vasculature is organized into lymphatic capillaries, pre-collecting, and collecting lymphatic vessels. The lymphatic capillaries have sparse basement membrane and consist of oak-leaf shaped lymphatic endothelial cells (LECs) characterized by the presence of “button-like” cell junctions formed by discontinuous adherens and tight junction protein complexes. Additionally, lymphatic capillaries are tightly connected to the extracellular matrix (ECM) via anchoring filaments. Local hydrostatic pressure then facilitates the opening of overlapping junctions by stretching anchoring filaments, which then promotes the uptake of interstitial fluid and migration of immune cells into the lymphatic vasculature. In contrast to capillaries, collecting vessel LECs function to transport lymph and exhibit continuous “zipper-like” cell junctions and are surrounded by basement membrane and lymphatic smooth muscle cells (blue). Collecting vessels are also arranged into lymphangion regions, separated by intraluminal lymphatic valves, which then help to maintain the unidirectional transport of lymph. The formation of valve LECs is driven by biomechanical transduction of exposure to oscillatory shear stress and valve LECs are attached to specialized ECM and lack lymphatic smooth muscle coverage. **(B)** Specialized lymphatic vessels, known as lacteals, are present in each villus of the small intestine. Lacteals are blind-ended lymphatic vessels consisting of LECs with “button-like” junctions surrounded by a blood vasculature capillary network and differentiated, specialized epithelial cells forming the villus. Lacteals function in the maintenance of intestinal homeostasis and gut immunity as well as digestive fat absorption. Specialized absorptive cells in the villus known as enterocytes are responsible for the uptake of fatty acids and monoglycerides from the intestinal lumen, which are then combined with proteins to form chylomicrons that are secreted and transported to the lacteals. Open regions of “button”-like junctions in the lacteals facilitate paracellular chylomicron transport into the lacteal lumen whereas transcellular chylomicron transport is mediated by pinocytic vesicle uptake and transport across the LEC cytoplasm into the lymphatic lumen. CM, chylomicron; JNC, junction; LEC, lymphatic endothelial cell; LL, lacteal lumen.

The development of the lymphatic vascular system in the mouse begins shortly after blood circulation is established (Escobedo and Oliver, 2016; Kazenwadel and Harvey, 2016; Semo et al., 2016). At approximately embryonic day (E) 9.5, a subpopulation of lymphatic endothelial progenitors located in the anterior cardinal vein become positive for Prospero-related homeobox 1 (Prox1) expression, which is the master regulator of the lymphatic vascular phenotype (Wigle and Oliver, 1999; Francois et al., 2008), and competent for differentiation into lymphatic endothelial cells (LECs) (Lee et al., 2009; Yamazaki et al., 2009; Srinivasan et al., 2010; Srinivasan and Oliver, 2011; Aranguren et al., 2013). Once LEC identity is specified (at ~E10.0), Prox1+ lymphatic endothelial progenitors that express the vascular endothelial growth factor receptor 3 (VEGFR-3) bud off via stimulation by the VEGF-C ligand derived from the mesenchyme and migrate dorsolaterally from the cardinal and intersomitic veins, creating chains of interconnected cells that subsequently form the primary lymph sacs and superficial lymphatic vessels (Karkkainen et al., 2004; Francois et al., 2012; Yang et al., 2012; Hagerling et al., 2013). Recent evidence also indicates that non-venous derived lymphatic precursors contribute to the lymphatic vasculature in the developing skin, heart, and mesentery (Klotz et al., 2015; Martinez-Corral et al., 2015; Stanczuk et al., 2015). These primary lymphatic structures develop into the lymphatic vascular network through the proliferation, sprouting, and survival of LECs, and this process is regulated by lymphangiogenic signaling such as the VEGF-C/D-VEGFR-3 and Angiopoietin (Angpt)-TEK (Tie2) pathways (Potente and Makinen, 2017). By E14.5, the network extends throughout the mouse embryo (Coso et al., 2014); then, beginning at E15.5-E16.0, the primary lymphatic vasculature undergoes remodeling and maturation to form a hierarchical lymphatic vascular network composed of lymphatic capillaries, precollecting and collecting lymphatic vessels (Figure 1A). The morphological changes associated with lymphatic remodeling and maturation also continue after birth.

## Formation and Regulation of Specialized Lymphatic Endothelial Cell Junctions

Lymphatic capillaries (also called initial lymphatics) are blind-ended and highly permeable, because their basement membrane is discontinuous, and they are not covered by pericytes or lymphatic smooth muscle cells. Furthermore, lymphatic capillaries are joined by discontinuous, button-like junctions at the border of oak leaf-shaped endothelial cells as they take up interstitial fluid and serve as entrance points for immune cells that infiltrate from blood vessels (Figure 1A). Such specialized button-like junctions are present in many tissues, including the trachea, diaphragm, dermis, and small intestine (Baluk et al., 2007; Dejana et al., 2009a; Yao et al., 2012; Bernier-Latmani and Petrova, 2017; Duong and Vestweber, 2020; Zhang et al., 2020). In contrast, continuous, zipper-like junctions are formed in the endothelium of collecting lymphatic vessels, which are surrounded by intact basement membranes and lymphatic smooth muscle cells and contain intraluminal valves. Collecting vessels then transport lymph to the circulatory system via lymph nodes (Martinez-Corral and Makinen, 2013; Petrova and Koh, 2018).

During mouse embryonic development, continuous zipper-like lymphatic junctions are initially formed in Prox1+ cells budding from the cardinal vein and in the jugular lymph sacs at E12.5 as well as in the tracheal lymphatic plexus at E 16.5 (Yao et al., 2012). The transformation to button-like junctions in the initial lymphatics of the trachea and diaphragm begins at E17.5 before birth and is mostly complete by postnatal day (P) 28 (Yao et al., 2012). In contrast, collecting lymphatic vessels maintain zipper-like junctions. While initial lymphatics of P4 neonatal mice contain intermediate cell-junctions between zippers and buttons, treatment with dexamethasone, an anti-inflammatory corticosteroid, promotes button junction formation (Yao et al., 2012). Sustained inflammation by *Mycoplasma pulmonis* infection in the respiratory tract induces button-to-zipper junctional conversion in the existing initial lymphatics of the trachea (Yao et al., 2012), whereas zipper-like junctions are present in growing tips of lymphatic sprouts in the tracheal mucosa (Baluk et al., 2007). Similar to neonatal lymphatics, the button-to-zipper transformation in inflammation can be reversed by the treatment of dexamethasone (Yao et al., 2012). These findings indicate the plasticity of LEC junction integrity between zippers and buttons both in development and inflammation.

The integrity of LEC junctions is tightly regulated by cell junction molecular complexes. Particular attention has been paid for the role of the adherens junction molecule vascular endothelial (VE)-cadherin in lymphatic junction stability in different tissues/organs such as mesenteric and dermal lymphatics, lacteals, and lymphatic valves (Hagerling et al., 2018; Yang et al., 2019; Duong and Vestweber, 2020; Petrova and Koh, 2020; Zhang et al., 2020). VE-cadherin is present in both button- and zipper-like junctions in the lymphatic endothelium, whereas its localization is particularly restricted in buttons at LEC borders (Baluk et al., 2007; Yao et al., 2012). Endothelial adhesion is stabilized by anchoring the VE-cadherin cytoplasmic tail to the actin cytoskeleton. In the blood endothelium, VEGF-A/VEGFR-2-mediated activation of the small GTPase RhoA/Rho-associated protein kinase (ROCK) signaling leads to cytoskeletal rearrangement of cortical actin into perpendicular stress fibers binding to VE-cadherin, thereby regulating cell junctions and vascular permeability (Dejana et al., 2009b; Dorland and Huveneers, 2017; Szymborska and Gerhardt, 2018). As described below, accumulating evidence suggest that the RhoA/ROCK pathway controls LEC junction integrity (Zhang et al., 2018; Frye et al., 2020; Norden et al., 2020).

## Angiopoietin 2-Dependent Formation of Button-Like Junctions in Lymphatic Capillaries

The Angiopoietin (Angpt)/TEK (Tie2) signaling pathway, which controls blood vessel stability and remodeling, is also required for the formation and maintenance of lymphatic vessels (Eklund et al., 2017; Saharinen et al., 2017; Akwii et al., 2019; Petrova and Koh, 2020). While the function of the Angpt2 ligand in blood vessels is context-dependent, it acts as an agonist for the TEK receptor in LECs during lymphangiogenesis (Gale et al., 2002; Dellinger et al., 2008; Shen et al., 2014; Yuen et al., 2014; Zheng et al., 2014; Souma et al., 2018). Notably, Angpt2 regulates the transformation of zipper-like junctions into button-like junctions during lymphatic vessel development (Zheng et al., 2014) (Table 1). The treatment of an Angpt2 blocking antibody does not affect zipper-like patterns in the sprouting front of lymphatic vessels of the mouse embryonic skin, whereas it blocks the transformation into button-like junctions in the plexus behind the sprouts in initial lymphatics, accompanied by a defect in phosphorylation of VE-cadherin that is associated with destabilization of cell-cell junctions. Dexamethasone-mediated induction of button-like junction formation in the sprouting vessel front, as well as the following plexus, is also inhibited by the Angpt2 blocking antibody. However, Angpt2 is only required for junction remodeling but is dispensable for the maintenance of button-like junctions of initial lymphatics. Similarly, the zipper-to-button junction transformation in initial lymphatics is inhibited in the neonatal mesentery and adult skin of *Angpt2* mutant mice (Zheng et al., 2014). Furthermore, both blocking antibody mediated inhibition of Angpt2 and genetic deletion of *Angpt2* lead to the disruption of LEC junctions in mesenteric collecting vessels leading to chyle leakage. This indicates that Angpt2 is also essential for the maintenance of junctional integrity in lymphatic collecting vessels.

**Table 1:** Factors involved in regulating lymphatic cell junctions.

**Factor**	**Function**	**References**
Angpt2	Essential for the formation of button-like junction in initial lymphatics	Zheng et al., 2014
	Maintains junctional integrity of collecting vessels	
VEGF-C	Secreted from villus macrophages	Suh et al., 2019
	VEGF-C/VEGFR-3 signaling controls button junctions in lacteals	
VEGFR-2	VEGF-A/VEGFR-2 signaling regulates zipper junctions in lacteals	Zhang et al., 2018
DLL4	Essential for lacteal regeneration via maintaining button junctions	Bernier-Latmani et al., 2015
	Downstream of VEGFR-2/3	
Calcrl	Upstream of Notch/DLL4 signaling in lacteal junctional integrity	Davis et al., 2017
	Controls the transcellular and paracellular transport pathways of chylomicrons	Davis et al., 2019
FOXC1/FOXC2	Required for LEC junction integrity in lymphatic valves, collecting vessels, and dermal lymphatics	Norden et al., 2020 Fatima et al., 2016
S1PR1/LPAR1	Crosstalk between LPAR1 and S1PR1 promotes porous LEC junctions of lymph nodes	Hisano et al., 2019
EphrinB2/EphB4	Maintains LEC junctions via RhoA-dependent cytoskeletal organization	Frye et al., 2020
RhoA/ROCK	Essential for LEC junction formation in lacteals, lymphatic valves and collecting vessels	Zhang et al., 2018 Norden et al., 2020
	Involved in EphrinB2/EphB4- and S1P/S1PR1-dependent junctional/cytoskeletal changes	Frye et al., 2020 Geng et al., 2020

## Key Signaling Pathways that Regulate Lacteal Junctions in the Small Intestine

Lacteals are blunt-ended, tube-like lymphatic capillaries in small intestinal villi which are essential for dietary fat absorption, gut immunity, and intestinal fluid homeostasis. Lacteal endothelial cells contain a mix of button-like and zipper-like junctions (Bernier-Latmani and Petrova, 2017; Petrova and Koh, 2018, 2020; Cifarelli and Eichmann, 2019) (Figure 1B). Here, dietary lipids are packaged into chylomicrons in enterocytes of the intestinal epithelium, and chylomicron entry into the lacteal lumen is thought to be mediated by paracellular transport through open button-like junctions (Casley-Smith, 1962; Sabesin and Frase, 1977; Bernier-Latmani et al., 2015; Zhang et al., 2018), although other studies have shown a transcellular transport mechanism (Dixon et al., 2009; Dixon, 2010; Reed et al., 2013). The formation and function of intestinal lacteals, including LEC junctions, are tightly regulated by several signaling pathways. The initial development of intestinal lymphatic vessels is dependent on activation of the VEGF-C/VEGFR-3/phosphatidylinositol 3-kinase (PI3K) pathway (Kim et al., 2007; Stanczuk et al., 2015). Unlike quiescent lymphatic vessels located in other adult tissues, lacteals are continuously maintained in a regenerative, slowly proliferative state undergoing lymphangiogenesis through the VEGF-C/VEGFR-3 pathway (Bernier-Latmani et al., 2015; Nurmi et al., 2015), which is attributable to the constant regeneration of the intestinal structure, including the intestinal epithelium containing stem cell populations, in order to maintain gut homeostasis (Barker, 2014). Moreover, intestinal villus SMCs and macrophages are reported to produce VEGF-C to regulate lacteal maintenance (Nurmi et al., 2015; Suh et al., 2019).

The continuous regeneration of lacteals is also regulated by Notch signaling. Expression of the Notch ligand Delta-like ligand 4 (DLL4) in lacteals is mediated by activation of VEGFR-2 and VEGFR-3 signaling, and LEC-specific deletion of *Dll4* in mice results in lacteal regression, reduced button-like junction formation, and impaired dietary fat uptake (Bernier-Latmani et al., 2015). Thus, Notch/DLL4 signaling is critical for lacteal maintenance and junctional integrity (Table 1). DLL4 expression in lacteals is also controlled by the peptide hormone adrenomedullin (AM) and its receptor, calcitonin receptor–like receptor (gene = *CALCRL*, protein = CLR) (Davis et al., 2017). Consistent with evidence that mutations in *Calcrl* are associated with autosomal recessive non-immune hydrops fetalis with lymphatic dysplasia in humans (Mackie et al., 2018), global deletion of *Calcrl* in mice causes systemic lymphatic insufficiency and lymphangiectasia (Hoopes et al., 2012). LEC-specific *Calcrl* mutant mice exhibit small intestinal lymphangiectasia, characterized by dilated lacteals and protein-losing enteropathy (Davis et al., 2017). Importantly, *Calcrl-*mutant lacteals contain more continuous cell junctions with reduced expression of DLL4 compared to controls (Davis et al., 2017) (Table 1). Indomethacin challenge to induce severe enteropathy that recapitulates human Crohn's disease leads to impaired lipid uptake and junctional barriers in the intestine of LEC-specific *Calcrl* mutant mice. Recent evidence also indicates that CLR signaling controls the critical balance between transcellular and paracellular transport pathways of lipids in lacteals (Davis et al., 2019). Collectively, these studies identify molecular interactions involving the VEGF-C/VEGFR-3, Notch/DLL4, and AM/CLR pathways in intestinal lacteal regeneration, integrity, and function (Table 1).

**Figure 2:**
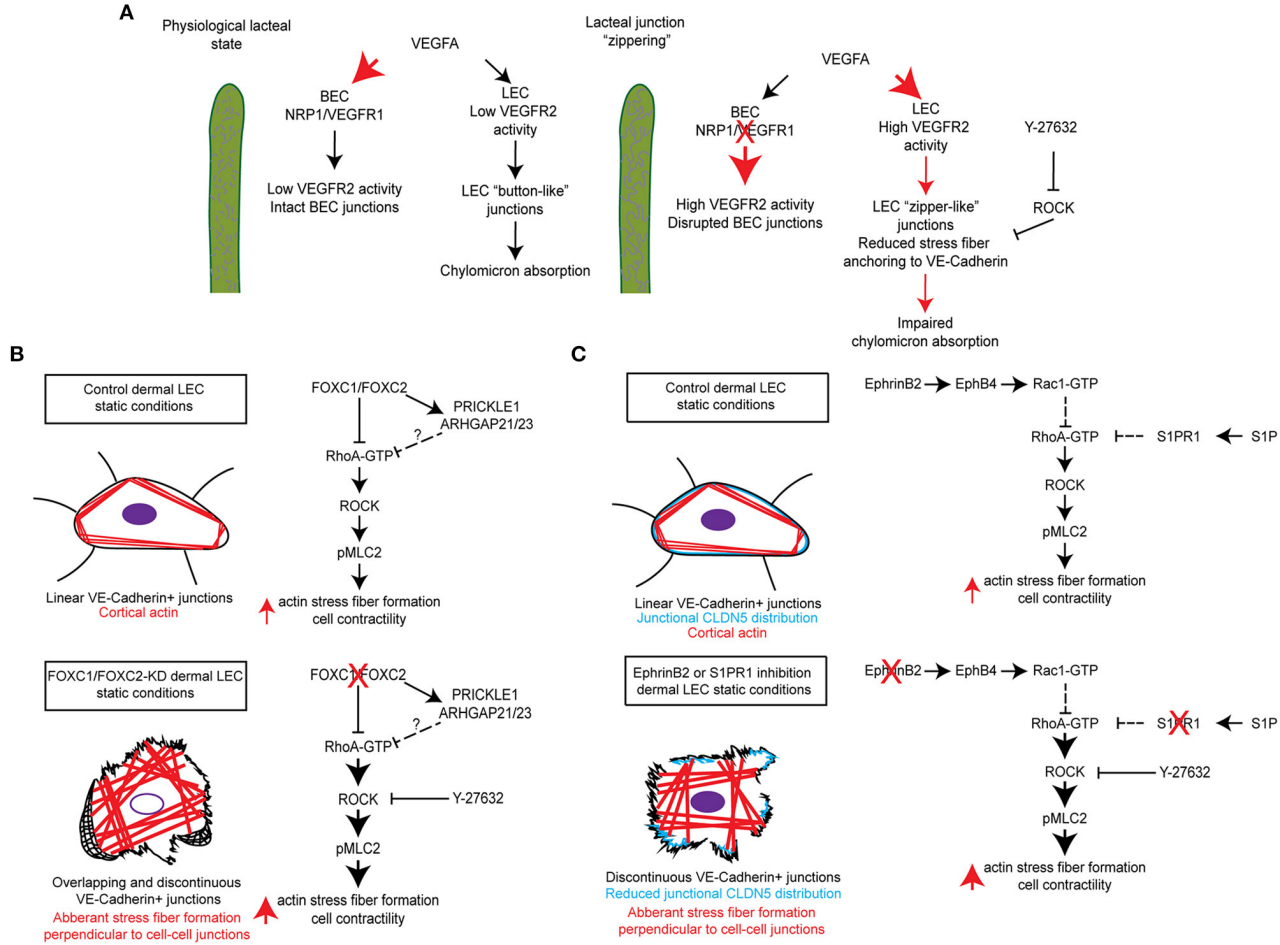
Molecular mechanisms regulating RhoA/ROCK signaling and cytoskeletal activity in maintenance of lymphatic junctional integrity. **(A)** In the lacteals, blood endothelial cell (BEC) expression of the receptors NRP1 and VEGFR-1 regulates the bioavailability of VEGFA resulting in low VEGFR-2 signaling activity in BECs, which maintains intact BEC junctions, and low VEGFR-2 signaling activity in LECs, which maintains “button-like” junctions and facilitates chylomicron absorption. Inducible endothelial-specific deletion of NRP1 and VEGFR-1 results in high VEGFA bioavailability and activation of BEC and LEC VEGFR-2, which results in disrupted BEC junctions and the formation of “zipper-like” junctions in LECs by reducing actin stress fiber anchoring to VE-Cadherin. This in turn impairs chylomicron absorption into lacteals. Moreover, chemical inhibition of ROCK by Y-27632 also reduces stress fiber anchoring to VE-Cadherin and induces the formation of LEC “zipper-like” junctions under physiological conditions (Zhang et al., 2018). **(B)** siRNA-mediated knockdown of FOXC1 and FOXC2 in human dermal lymphatic endothelial cells (HDLECs) induced the formation of overlapping and discontinuous VE-Cadherin+ cell junctions and stimulated aberrant actin stress fiber formation perpendicular to VE-Cadherin+ cell junctions. Treatment of cells with the ROCK inhibitor Y-27632 was able to rescue this impaired phenotype and restore continuous junctions in HDLECs. It is postulated that transcriptional regulation of a molecular signaling complex, consisting of the planar cell polarity signaling component PRICKLE1 and the RhoA GTPase activating proteins (GAPs) Arhgap21 and Arhgap23, by FOXC1 and FOXC2 negatively regulates downstream Rho/ROCK signaling as previously described (Norden et al., 2020). **(C)** siRNA-mediated knockdown of the ligand EphrinB2, subsequently resulting in impaired signaling through its receptor EphB4, was shown to reduce Rac1 GTPase activation in HDLECs. Furthermore, antibody mediated inhibition of EphrinB2 resulted in the formation of discontinuous cell junctions, which was accompanied by reduced junctional CLDN5 distribution and stimulation of aberrant actin stress fiber formation (Frye et al., 2020). In contrast, siRNA-mediated knockdown or chemical inhibition of S1PR1 in HDLECs was shown to enhance RhoA GTPase activation, which also resulted in the formation of discontinuous cell junctions and reduced junctional CLDN5 distribution (Geng et al., 2020). In both instances, pretreatment of HDLECs with Y-27632 was able to rescue the impaired phenotype induced by inhibition of either EphrinB2/EphB4 or S1PR1 signaling.

Lacteals are surrounded by villus blood capillaries. Recent studies demonstrate that the close localization of the intestinal blood and lymphatic vessels is important to maintain the junctional integrity and dietary fat uptake of lacteals. Within the villi, the bioavailability of VEGF-A is restricted by VEGFR-1 and NRP1, both of which are highly expressed on blood capillaries, but not on lacteals (Zhang et al., 2018). As decoy receptors, they bind VEGF-A and compete for VEGFR-2. Lack of VEGFR-1 and NPR1 in mice increases VEGF-A availability in the villi, which in turn activates VEGFR-2 signaling in lacteals and induces the button-to-zipper junctional transformation, thereby preventing chylomicron uptake into lacteals. Such lacteal junction zippering protects mice from high-fat diet-induced obesity (Zhang et al., 2018) (Figure 2A). In contrast, high VEGF-A bioavailability disrupts cell junctions of villus blood capillaries. This discrepancy between lacteals and villus blood capillaries appears to be associated with VEGFR-2-dependent activation of RhoA/ROCK/phosphorylated myosin light chain (pMLC) signaling in the regulation of cytoskeletal organization (Ridley, 2001; Hall, 2012; Knipe et al., 2015). The RhoA/ROCK pathway regulates stress fiber formation and focal adhesion dynamics in blood endothelial cell barrier function and permeability (Carbajal et al., 2000; Van Nieuw Amerongen et al., 2000; Wojciak-Stothard et al., 2001; Spindler et al., 2010; Bowers et al., 2016; Cerutti and Ridley, 2017). As endothelial junctions are maintained by a balance of the actin cytoskeleton and myosin-based actin pulling forces anchoring to endothelial junctions, RhoA/ROCK-dependent cytoskeletal dynamics controls endothelial junction integrity (Dorland and Huveneers, 2017). In cultured LECs, VEGFR-2 activation induces junction zippering by reducing actin stress fiber anchoring to perpendicularly formed VE-cadherin, which is attributable to the inhibition of ROCK activity (Zhang et al., 2018). Furthermore, treatment of neonatal mice with the ROCK inhibitor Y27632 enhances lacteal zipper junction formation and reduces chylomicron transport into mesenteric lymphatic vessels, whereas the ROCK inhibition does not affect junctions of villus blood capillaries (Zhang et al., 2018) (Table 1, Figure 2A). Considering these differences in the vasculature of the villi, further studies are needed to elucidate the molecular mechanisms underlying opposing effects of VEGFR-2 signaling on blood and lymphatic endothelial cell junctions.

## FOXC1 and FOXC2 Transcription Factors as Regulators of LEC Junctional Integrity

FOXC1 and FOXC2 are closely related members of the FOX transcription factor family and have numerous essential roles in cardiovascular development, health, and disease (De Val and Black, 2009; Kume, 2009; Lam et al., 2013). Mutations or changes in the copy number of human *FOXC1* are associated with autosomal-dominant Axenfeld-Rieger syndrome, which is characterized by anterior segment abnormalities in the eye and extraocular defects (Tumer and Bach-Holm, 2009; Seifi and Walter, 2018), while inactivating mutations in human *FOXC2* are responsible for the autosomal dominant syndrome Lymphedema-distichiasis, which is characterized by obstructed lymph drainage in the limbs, venous valve failure, and by the growth of extra eyelashes (distichiasis) that arise from the meibomian glands (Mansour et al., 1993; Fang et al., 2000). FOXC2 expression is upregulated in LECs by oscillatory shear stress (OSS) and is highly enriched in the valve sinuses of lymphatic collecting vessels (Sabine et al., 2012, 2015). FOXC2 is an essential regulator of connexin 37 (Cx37) and calcineurin/NFAT signaling during lymphatic valve initiation (Petrova et al., 2004; Norrmen et al., 2009; Sabine et al., 2012) and is critical for the maintenance of lymphatic valves via regulation of LEC junctional integrity (Sabine et al., 2015). Corresponding with evidence that individuals with *FOXC2* mutations have hyperplastic lymphatic vessels (Mansour et al., 1993; Brice et al., 2002), FOXC2 as well as FOXC1 are essential negative regulators of developmental lymphangiogenesis (Fatima et al., 2016). Of note, dermal LEC junctions stained with VE-cadherin and Lyve1 are disrupted in the dorsal embryonic skin of E14.5 LEC-specific double mutant mice for *Foxc1* and *Foxc2*, suggesting that lack of the two genes impairs the junctional integrity of dermal lymphatic vessels (Fatima et al., 2016).

A recent study further demonstrates a complementary role of FOXC1 in addition to FOXC2 as key mediators of mechanotransduction in the regulation of LEC junctional integrity (Norden et al., 2020). Unlike FOXC2, FOXC1 is not increased in LECs by OSS, but by laminar shear stress (LSS), and FOXC1 is highly enriched in LECs located at the leading free-edge of the intraluminal side of valve leaflets that are exposed to LSS in mesenteric lymphatic valves of the adult mice (Norden et al., 2020). Inducible endothelial cell (EC)-specific *Foxc1* deletion in mice impairs postnatal lymphatic valve maturation, whereas EC-deletion of *Foxc2* induces valve degeneration, which is exacerbated in EC-specific compound *Foxc1* and *Foxc2* mutant mice. Mechanistically, *in vitro* loss of FOXC1 or FOXC2 induces hyper-activation of contractile stress fibers in LECs, which is rescued by the ROCK inhibitor Y27632. Pharmacological inhibition of ROCK by the treatment with Y27632 also improves LEC barrier integrity of mesenteric collecting vessels in both single EC-*Foxc2* and compound EC-*Foxc1; Foxc2* mutant mice, while valve degeneration is partially rescued in only EC-*Foxc2* mutants (Norden et al., 2020). These findings elucidate a key contribution of FOXC1 and FOXC2 in regulating lymphatic valve maintenance and LEC junction integrity via RhoA/ROCK-dependent cytoskeletal organization (Table 1, Figure 2B).

## Receptor Crosstalk of Lysophospholipids, Sphingosine 1-Phosphate (S1P) and Lysophosphatidic Acid (LPA)

S1P and LPA, structurally related lipid mediators, activate G protein–coupled receptors (GPCRs) to regulate various cellular processes, including cytoskeletal dynamics (Moolenaar and Hla, 2012; Proia and Hla, 2015). Despite evidence that the two lysophospholipids have redundant functions, the precise mechanisms of crosstalk between the S1P and LPA signaling pathways remain poorly understood. A genome-wide CRISPR/dCas9–based GPCR signaling screen recently identified the LPAR1 receptor as a key regulator of the S1PR1 receptor-mediated signaling coupling to the antagonistic, β-arrestin-dependent receptor internalization pathway (Hisano et al., 2019). High resolution imaging of cell-cell junctions of sinus-lining LECs of mouse lumbar, popliteal, brachial, and mesenteric lymph nodes reveals both continuous and punctate VE-cadherin+ LEC junctions. In mice treated with the LPAR1 inhibitor AM095 or in *Lpar1* mutant mice, S1PR1 coupling to β-arrestin is suppressed in sinus-lining LECs of lymph nodes, accompanied by a decrease in punctate junctions and an increase in continuous junctions. These findings suggest that LPAR1 signaling attenuates S1P signaling and enhances the junctional porosity of sinus LECs by suppressing the formation of continuous junctions. As LPAR1 signaling controls RhoA/ROCK-mediated cytoskeletal dynamics (Ridley, 2001; Hall, 2012; Knipe et al., 2015), LPA treatment induces stress fiber formation, increased phosphorylation of MLC, and the formation of punctate, intracellular gaps in VE-cadherin-stained cell junctions in human umbilical vein endothelial cells (HUVECs), whereas S1PR1 activation stimulates continuous, zipper-like junctions with cortical F-actin formation. Moreover, it was recently shown that S1PR1 signaling suppresses RhoA GTPase activation in cultured LECs, whereas its blockade results in the formation of discontinuous LEC junctions, which is rescued by ROCK inhibitor Y-27632 (Geng et al., 2020) (Figure 2C). In contrast to activation of LPAR1 or S1PR1 individually in HUVECs, combined activation of both LPAR1 and S1PR1 results in a hybrid of continuous cell junctions interspersed with punctate VE-cadherin+ structures at the termini of actin-rich stress fibers. Furthermore, LPAR1 signaling attenuates S1PR1-induced barrier function *in vivo*. In mice treated with the LPAR1 inhibitor AM095, the retention of lymphocytes in lymph nodes is enhanced, suggesting that LPAR1 signaling is critical for the regulation of lymphatic sinus junctional porosity. Together, these results indicate that the crosstalk of S1PR1 and LPAR1 signaling regulates the junctional architecture, barrier function, and lymphocyte egress in sinus-lining LECs of lymph nodes (Hisano et al., 2019) (Table 1). Additionally, S1P signaling regulates the formation of button-like junctions of the diaphragm initial lymphatics (Pham et al., 2010). However, whether there is a similar crosstalk mechanism between S1PR1 and LPAR1 in the initial lymphatics remains to be investigated.

## The Ephrinb2-Ephb4 Signaling Pathway in LEC Junction Stability

The transmembrane ligand EphrinB2 and its receptor EphB4 are essential for both blood and lymphatic vessel development (Adams et al., 1999; Gerety et al., 1999; Makinen et al., 2005; Zhang et al., 2015; Yoshimatsu et al., 2020). However, the role of this signaling pathway in vessel integrity remains largely unknown. Frye et al. shows that postnatal EC-deletion of *EphrinB2* or *EphB4* in mice results in disruption of cell junctions in different lymphatic vascular beds, including dermal and mesenteric collecting lymphatic vessels as well as the subcapsular sinus of the inguinal lymph nodes, whereas the EphrinB2/EphB4 pathway is dispensable for blood vessel integrity (Frye et al., 2020). *In vitro* studies reveal that inhibition of EphrinB2 by a blocking antibody in LECs causes disruption of cortical actin along with an increase in central actin (radial actin and actin stress fibers) and monolayer permeability. As increased actin stress fibers are associated with enhanced RhoA activity, which is negatively regulated by Rac1 (Wu et al., 2009), inhibition of EphB4 activity by Ephrin B2 knockdown in LECs reduces Rac1 activity, thereby increasing RhoA activity. Furthermore, pretreatment with the ROCK inhibitor Y-27632 inhibits the EphrinB2 blockade-induced junctional and cytoskeletal effects in LECs. Together, basal EphrinB2/EphB4 signaling controls the stability of LEC junctions via RhoA/ROCK-dependent regulation of cytoskeletal contractility (Frye et al., 2020) (Table 1, Figure 2C).

## Concluding Remarks

Given the plasticity of specialized lymphatic endothelial cell junctions, zipper- and button-like junctions are tightly maintained to keep lymphatic vessel integrity and function for tissue homeostasis. It has become increasingly evident that disrupted LEC junctions are potentially associated with various diseases, including lymphatic leakage present in chylothorax and lymphedema, metabolic syndrome, and impaired immune surveillance (Cifarelli and Eichmann, 2019; Jiang et al., 2019; Xiao et al., 2019; Norden and Kume, 2020; Zhang et al., 2020). The recent seminal studies summarized in this review provide compelling evidence that RhoA/ROCK signaling regulates LEC junction integrity in different vascular beds (Zhang et al., 2018; Hisano et al., 2019; Frye et al., 2020; Norden et al., 2020) (Table 1, Figure 2). LECs are known to have the ability to undergo endothelial-to-mesenchymal transition (EndMT) by acquiring a mesenchymal cell phenotype, including loss of cell-cell junctions (Ichise et al., 2014; Dejana et al., 2017; Yoshimatsu et al., 2020). Another recent study demonstrates that as a non-Smad pathway, RhoA/ROCK signaling participates in TGF-β-induced EndMT of human dermal LECs *in vitro* (Yoshimatsu et al., 2020). This observation also reinforces the importance of RhoA/ROCK activity in the regulation of LEC junction integrity. As shown in rodent models (Zhang et al., 2018), selective targeting RhoA/ROCK signaling in lacteals of the small intestine is clinically of significance to a novel therapeutic approach for the treatment of obesity and metabolic dysfunction. ROCK inhibitors have been shown to have beneficial effects in experimental animal models of cardiovascular and metabolic disease (Kikuchi et al., 2007; Shi and Wei, 2013; Okin and Medzhitov, 2016), as well as for treatment of cerebral vasospasm, a condition in which the blood vessels in the brain narrow and blood flow is reduced (Shibuya et al., 1992; Zhao et al., 2006; Liu et al., 2012). Yet, additional comprehensive studies are needed to fully elucidate the mechanisms by which the signaling pathways associated with transcriptional regulation control LEC junction integrity. Investigations into the molecular and cellular mechanisms that support the formation, maintenance, and function of lymphatic vessels will have critical implications for the development and optimization of potential therapeutic targets to modulate LEC junctions, permeability and function in disease characterized by dysregulated inflammation, lipid metabolism, and immune responses.

## Author Contributions

PN contributed to the editing of the manuscript and making the figures. TK contributed to the concepts, writing, editing, and final formatting of the manuscript. All authors contributed to the article and approved the submitted version.

## Conflict of Interest

The authors declare that the research was conducted in the absence of any commercial or financial relationships that could be construed as a potential conflict of interest.
